# Mutation of *ATF6* causes autosomal recessive achromatopsia

**DOI:** 10.1007/s00439-015-1571-4

**Published:** 2015-06-11

**Authors:** Muhammad Ansar, Regie Lyn P. Santos-Cortez, Muhammad Arif Nadeem Saqib, Fareeha Zulfiqar, Kwanghyuk Lee, Naeem Mahmood Ashraf, Ehsan Ullah, Xin Wang, Sundus Sajid, Falak Sher Khan, Muhammad Amin-ud-Din, University of Washington Center for Mendelian Genomics, Joshua D. Smith, Jay Shendure, Michael J. Bamshad, Deborah A. Nickerson, Abdul Hameed, Saima Riazuddin, Zubair M. Ahmed, Wasim Ahmad, Suzanne M. Leal

**Affiliations:** Department of Molecular and Human Genetics, Center for Statistical Genetics, Baylor College of Medicine, 1 Baylor Plaza 700D, Houston, TX 77030 USA; Department of Biochemistry, Faculty of Biological Sciences, Quaid-i-Azam University, Islamabad, 45320 Pakistan; Department of Otorhinolaryngology Head and Neck Surgery, School of Medicine, University of Maryland, Baltimore, MD 21203 USA; University of Education, Dera Ghazi Khan Campus, Lahore, 32200 Pakistan; Department of Genome Sciences, University of Washington, Seattle, WA 98195 USA; Institute of Biomedical and Genetic Engineering, Islamabad, 44000 Pakistan

## Abstract

Achromatopsia (ACHM) is an early-onset retinal dystrophy characterized by photophobia, nystagmus, color blindness and severely reduced visual acuity. Currently mutations in five genes *CNGA3*, *CNGB3*, *GNAT2*, *PDE6C* and *PDE6H* have been implicated in ACHM. We performed homozygosity mapping and linkage analysis in a consanguineous Pakistani ACHM family and mapped the locus to a 15.12-Mb region on chromosome 1q23.1–q24.3 with a maximum LOD score of 3.6. A DNA sample from an affected family member underwent exome sequencing. Within the *ATF6* gene, a single-base insertion variant c.355_356dupG (p.Glu119Glyfs*8) was identified, which completely segregates with the ACHM phenotype within the family. The frameshift variant was absent in public variant databases, in 130 exomes from unrelated Pakistani individuals, and in 235 ethnically matched controls. The variant is predicted to result in a truncated protein that lacks the DNA binding and transmembrane domains and therefore affects the function of ATF6 as a transcription factor that initiates the unfolded protein response during endoplasmic reticulum (ER) stress. Immunolabeling with anti-ATF6 antibodies showed localization throughout the mouse neuronal retina, including retinal pigment epithelium, photoreceptor cells, inner nuclear layer, inner and outer plexiform layers, with a more prominent signal in retinal ganglion cells. In contrast to cytoplasmic expression of wild-type protein, in heterologous cells ATF6 protein with the p.Glu119Glyfs*8 variant is mainly confined to the nucleus. Our results imply that response to ER stress as mediated by the ATF6 pathway is essential for color vision in humans.

**Electronic supplementary material:**

The online version of this article (doi:10.1007/s00439-015-1571-4) contains supplementary material, which is available to authorized users.

## Introduction

Achromatopsia (ACHM) is an autosomal recessive heterogeneous disorder characterized by symptoms that include the inability to discriminate colors, reduced visual acuity primarily in daylight, nystagmus and severe photophobia (Michaelides et al. 2004). Initially, ACHM was considered as a stationary disorder but recent imaging studies have shown the progressive nature of disease in several patients (Thiadens et al. 2010; Aboshiha et al. 2014). Its prevalence is estimated to be about 1 in 30,000 individuals worldwide. To date, mutations in *CNGA3* [MIM 600053], *CNGB3* [MIM 605080], *GNAT2* [MIM 139340], *PDE6C* [MIM 600827] and *PDE6H* [MIM 601190] are known to cause ACHM (Kohl et al. 1998, 2000, 2002, 2012; Chang et al. 2009). The proteins encoded by these genes are cone-specific and have critical roles in the phototransduction cascade occurring in the cone photoreceptors. *CNGA3* and *CNGB3* mutations are the major cause of ACHM worldwide, with mutations in *GNAT2, PDE6C* and *PDE6H* playing a lesser role (Kohl et al. 2012). However, the known gene mutations do not account for all cases of ACHM (Saqib et al. 2011).

Using homozygosity mapping and linkage analysis, we mapped a new locus for ACHM in a consanguineous Pakistani family. Within the mapped region, from exome sequence data we identified a frameshift variant c.355_356dupG (p.Glu119Glyfs*8) in the *ATF6* [MIM 605537] gene which encodes cyclic AMP-dependent activating transcription factor-6 alpha. The p.Glu119Glyfs*8 variant affects the targeting of ATF6 protein in heterologous cells. Immunostaining revealed ubiquitous expression of ATF6 in the neuronal retina. Our results highlight the importance of the ATF6-mediated signaling pathway to color vision in humans.

## Materials and methods

### Subjects

**Fig. 1:**
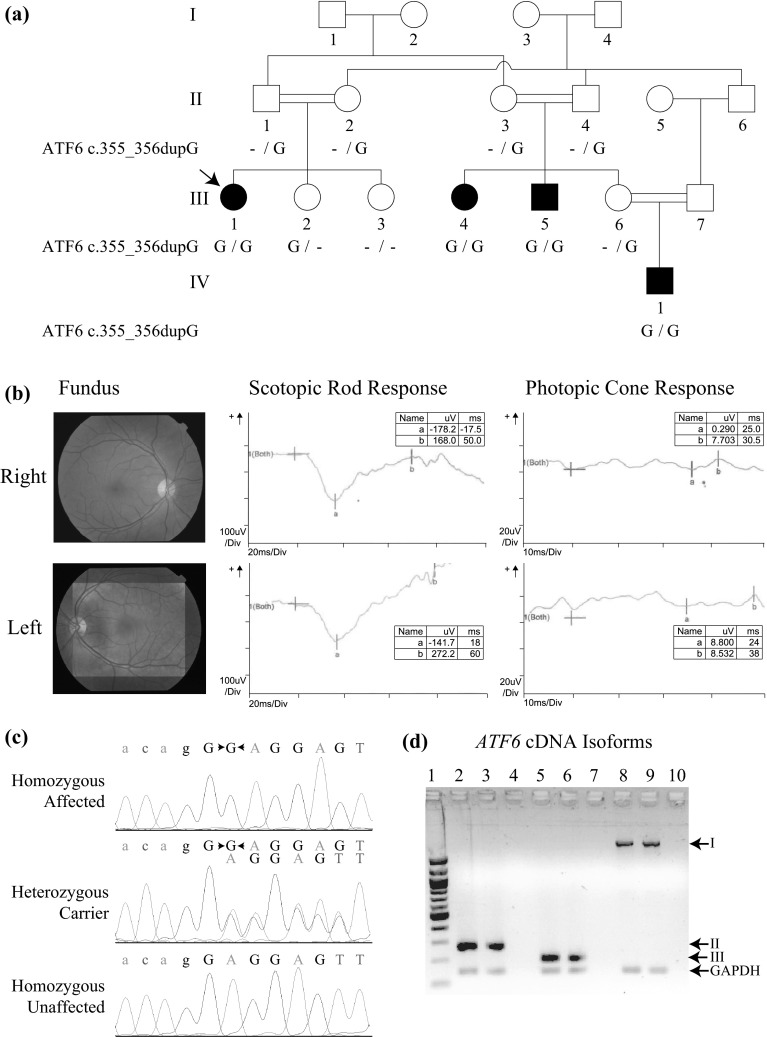
Pedigree drawing, clinical findings, chromatograms and RT-PCR expression results for *ATF6.* **a** Pedigree drawing of MA28 family with autosomal recessive ACHM. Genotypes for the *ATF6* c.355_356dupG variant are shown below each symbol of corresponding family members with available DNA samples. **b** Fundoscopy images and ERG data for affected proband III-1 (*arrow* in *panel* **a**) which shows bilateral loss of the foveal reflex and reduced cone response, respectively. Other retinal features are normal, including a pale-centered optic disc with clear borders, 0.3–0.4 cup-to-disc ratio, and no arteriovenous nicking, hemorrhages, emboli or infarcts. **c** Chromatograms of family members who are homozygous, heterozygous and wild-type for the *ATF6* variant. **d** RT-PCR results showed the presence of multiple *ATF6* isoforms in human eye and retinal pigment epithelium cells.* I* NM_007348.3 exons 1–16, 2028 bp;* II* NM_007348.3 exons 15–16, 265 bp;* III* AB208929 exon 14, 190 bp. Table S1 has additional details on isoforms and exons tested. GAPDH (143 bp) was used as internal control. *Lanes: 1* 100-bp leader; *2*, *5*, *8* whole eye; *3*, *6*, *9* retinal pigment epithelium; *4*, *7*, *10* negative control

This study was initiated after obtaining approval from the institutional review boards of Quaid-i-Azam University and Baylor College of Medicine and Affiliated Hospitals. Informed consent was obtained from all participating individuals. Blood samples were collected from four affected and seven healthy individuals (Fig. 1a) and genomic DNA was extracted with GenElute™ blood genomic DNA kit (Sigma-Aldrich, St. Louis, MO, USA). A detailed interview was conducted with family members to gather information on pedigree structure, comorbidities, onset of disease and initial symptoms. The clinical diagnosis was based on presenting symptoms, physical examinations, visual acuity measurement, fundoscopy, color vision test and full-field electroretinography (ffERG).

### Genotyping and linkage analysis

DNA samples from 11 family members underwent a genome-wide scan using the Infinium^®^ HumanCoreExome BeadChip (Illumina, USA) which contains ~550 K single-nucleotide polymorphism (SNP) markers. The resulting genotype data were analyzed using PedCheck (O’Connell and Weeks 1998) to identify Mendelian inconsistences and MERLIN (Abecasis et al. 2002) to detect double recombination events, which occur within small physical distances that are most likely due to genotyping errors. Markers with suspected errors were removed from subsequent analyses. HomozygosityMapper (Seelow et al. 2009) was used to identify regions of homozygosity. Two-point and multipoint parametric linkage analyses were performed using Superlink (Silberstein et al. 2006) and MERLIN respectively, using a fully penetrant autosomal recessive mode of inheritance with no phenocopies and a disease allele frequency of 0.001. Allele frequencies for SNP markers were estimated from the founders and reconstructed founders from 16 Pakistani families for whom genotype data were obtained using the same array. For multipoint linkage analysis, genetic map positions were obtained through interpolation from physical maps to the Rutgers combined linkage-physical map build 37 (Matise et al. 2007).

### Exome sequencing

Exome sequencing was performed using a DNA sample from affected individual III-1 (Fig. 1a). Sequence capture was performed with the Roche NimbleGen SeqCap EZ Human Exome Library v.2.0 to target 36.6 Mb of sequence. Sequencing was carried out on an Illumina HiSeq and fastq files were aligned to the human reference sequence (hg19) using Burrows–Wheeler Aligner (Li and Durbin 2009). Realignment of regions containing indels, recalibration of base qualities, and variant detection and calling were carried out using the Genome Analysis Toolkit (McKenna et al. 2010). Annotation of variant sites was performed using SeattleSeq 137.

The exome data were analyzed to find within the region of homozygosity coding or splice variants which are not in dbSNP and had a minor allele frequency (MAF) <0.05 % in the Exome Aggregation Consortium (ExAC) database which includes data from >60,000 next-generation sequences. Occurrence of the *ATF6* c.355_356dupG variant was also examined using exomes from 130 unrelated Pakistani individuals who are affected with a Mendelian trait but do not have eye-related phenotypes.

### Mutation analysis

Sanger sequencing was used to confirm the co-segregation of the c.355_356dupG variant in exon 5 of *ATF6* (NM_007348.3) with ACHM within the family using primers 5′GGAAGAAATGTAGCAACAGATCAA3′ and 5′CCAGTGACAGGCTTATCTTCC3′. DNA samples from 235 Pakistani control individuals were also screened for the *ATF6* variant. Amplified PCR products were purified by ExoSAP-IT (Affymetrix, Cleveland, OH, USA) and sequenced using BigDye Terminator v3.1 Cycle Sequencing Kit (Life Technologies, Carlsbad, CA, USA). Sequence data were analyzed with Sequencher 4.9 (Gene Codes Corporation, Ann Arbor, MI, USA).

Multiple ATF6 and ATF6-like protein sequences were derived by blastp from the UniProt database and aligned using Clustal Omega (Sievers et al. 2011). Molecular modeling of wild-type and mutant ATF6 was performed using Phyre2 (Kelley and Sternberg 2009) with CREB protein structure as template (Protein Data Bank ID 1DH3).

### cDNA synthesis and reverse transcription (RT)-PCR

To check the expression of *ATF6* in the eye, total RNA samples were obtained for human eye (Biochain Institute, Newark, CA, USA) and retinal pigment epithelial cells (ScienCell Research Laboratories, Carlsbad, CA, USA). cDNA was synthesized using 1 μg RNA, SMARTScribe Reverse Transcriptase (Clontech, Mountain View, CA, USA) and random hexamers (Invitrogen, Carlslbad, CA, USA). Multiple primer sets (Table S1) were used to detect the expression of *ATF6* isoforms in the RNA samples from human eye and retinal pigment epithelium cells. *GAPDH* was used as internal control.

### Western blot analysis

All animals used in this study were kept with the approval of the Institutional Animal Care and Use Committee of the University of Maryland, School of Medicine. C57BL/6 mouse retinae were fixed in 4 % paraformaldehyde for 30 min, washed in PBS, and homogenized and sonicated in ice-cold RIPA lysis buffer (Teknova R3792, Hollister, CA, USA) supplemented with protease inhibitor cocktail (Sigma-Aldrich P8340, St. Louis, MO, USA). Proteins were extracted and denatured by boiling at 95 °C for 5 min in SDS-PAGE sample buffer (0.125 M Tris–HCl, 20 % glycerol, 4 % SDS, 0.005 % bromophenol blue). A 50-μg protein sample was separated on a 4–20 % gradient Tris–glycine gel (Novex, San Diego, CA, USA) and transferred to nitrocellulose membrane, blocked overnight at 4 °C with 5 % dry milk in TBST (10 mM Tris–HCl pH 7.5, 150 mM NaCl, 0.05 % Tween 20), and stained with anti-ATF6 antibodies (1:500 in blocking solution) for 1 h at room temperature. After three washes, the membranes were incubated in a 1:1500 dilution of horseradish peroxidase-conjugated anti-rabbit secondary antibody (GE Healthcare) for 1 h at room temperature and developed using the ECL Plus Western Blotting Detection system (Amersham Pharmacia Biotech, Arlington Heights, IL, USA).

We also performed western blot analysis to study the steady state level of wild-type and mutant ATF6 proteins in transfected COS-7 cells. After 24 h of transfection, COS-7 cells were collected and mixed with 2× sample loading buffer with reducing agent and processed for western blot analysis as described above.

### Immunostaining, expression plasmid and localization assay

Cryo-preserved retinal sections from adult CD1 mice were rehydrated in PBS with 0.1 % Triton X-100 for 20 min. After blocking in 10 % Normal Goat Serum (NGS) for 45 min at room temperature, sections were incubated with various concentrations of anti-ATF6 antibodies (abcam, Cambridge, MA, USA) in a dark humid chamber for 3 h, followed by washing in 1X PBS and conjugation with Alexa 488 secondary antibodies (Life Technologies, Carlsbad, CA, USA). For nuclear DNA, we used 4′6-diamidino-2-phenylindole (DAPI; 2 µg/ml). Actin was decorated with either rhodamine phalloidin (1:500) or Alexa 647-phalloidin (1:100). Sections were mounted with ProLong Gold antifade reagent (Molecular Probes, Life Technologies) and imaged using LSM510 (Zeiss, Pleasanton, CA, USA).

Human full-length (Accession no. NM_007348) *ATF6* flag-tagged and eGFP-tagged cDNA constructs were purchased from GeneCopoeia (Rockville, MD). To insert the c.355_356dupG mutation, we performed site-directed mutagenesis on the construct using the QuikChange Lightening Kit (Agilent Technologies, Santa Clara, CA). To study expression and targeting, we transfected COS-7 cells, grown in DMEM that was supplemented with 10 % FBS, with flag-tagged wild-type and mutant ATF6 constructs. Transfected cells were maintained at 37 °C in 5 % CO_2_ for 24 h. After incubation, transfected cells were fixed with 4 % paraformaldehyde (EMS) in PBS for 30 min, permeabilized for 15 min in 0.2 % Triton X-100 and nonspecific binding sites were blocked by incubation in 2 % BSA and 5 % normal goat serum in PBS for 30 min. COS-7 cells were incubated for 2 h at room temperature with anti-flag (1:500) and primary antibodies for Calregulin (1:500) as endoplasmic reticulum (ER) marker, followed by incubation with Alexa Fluor 488 goat anti-mouse and Alexa Fluor 546 goat anti-rabbit secondary antibodies (Molecular Probes) for 45 min at room temperature. Rhodamine phalloidin was used to label actin cytoskeleton, while nuclei were decorated with DAPI. Samples were washed in PBS, mounted using ProLong Gold Antifade reagent (Molecular Probes), and imaged with the use of a LSM510 coreduo confocal microscope equipped with a Zeiss 63X objective (Zeiss Microimaging).

### Antibody validation

To validate anti-ATF6 antibody (ab37149; abcam, Cambridge, MA, USA), we performed a colocalization assay using flag-tagged ATF6-transfected COS-7 cells. The cells were grown on coverslips in Dulbecco’s modified Eagle’s medium (Invitrogen) supplemented with 10 % fetal bovine serum and then transfected with Lipofectamine reagent (Invitrogen). After incubation at 37 °C with 5 % CO_2_ for 24 h, transfected cells were fixed with 4 % paraformaldehyde (EMS) in PBS for 30 min, permeabilized for 15 min in 0.2 % Triton X-100 and nonspecific binding sites were blocked by incubation in 2 % BSA and 5 % normal goat serum in PBS for 30 min. Primary ATF6 and anti-flag antibodies were diluted in blocking solution to a final concentration of ~0.5 μg per ml. COS-7 cells were incubated for 2 h at room temperature with ATF6 and anti-flag antibodies, followed by incubation with Alexa Fluor 546 goat anti-rabbit and Alexa Fluor 488 goat anti-mouse secondary antibodies (Molecular Probes) for 45 min at room temperature. Rhodamine phalloidin labeled the actin cytoskeleton and DAPI was used to stain nuclei. Samples were washed in PBS, mounted using ProLong Gold Antifade reagent (Molecular Probes), and imaged with the use of a LSM510 coreduo confocal microscope.

## Results

The four affected individuals (III-1, III-4, III-5, IV-1; Fig. 1a) of family MA28 presented with severe photophobia, nystagmus and absence of color discrimination from early childhood. They have normal night vision, but in daylight they experience extreme discomfort and exhibit extensive blinking. Complete physical examination and standard blood tests (e.g., blood cell counts, lipid profile, liver function tests, glucose, creatinine, among others) of all four affected individuals ruled out the presence of additional comorbidities (i.e., neurologic, otolaryngologic, hematologic, cardiovascular, respiratory, gastrointestinal, endocrine, urinary) and substantiated the presence of isolated achromatopsia. The visual acuity measurements of the affected individuals range from 0.08 to 0.2. The Ishihara 24-plate color test revealed severe defects in all affected individuals. Fundus examination of the proband III-1, at the age of 16 years, was normal except for loss of the foveal reflex (Fig. 1b). The ffERG of the same individual was typical of ACHM, i.e., (1) normal amplitude and latency of scotopic or rod response in dark-adapted phase at ≤30 Hz but subnormal rod response at 120 Hz, (2) absent photopic flicker response with loss of regular sinusoidal waveforms, and (3) normal latency but severely diminished amplitude of photopic or single cone flash response (Fig. 1b, S1). Additionally, oscillatory potentials were demonstrated during scotopic responses at 30 Hz (Fig. 1b, S1), presumably indicating amacrine cell function during the rod response.

Homozygosity mapping using genome-wide genotypes from 11 family members indicated a 15.12-Mb region of homozygosity on chromosome 1q23.1–q24.3 (Fig. S2a). Linkage analysis yielded a maximum two-point LOD score of 3.1 (*θ* = 0) at rs6700867 (chr1:162.31 Mb; NCBI Build hg19). A statistically significant maximum multipoint LOD score of 3.6 was obtained at several markers from rs1538971 (chr1:161.68 Mb) to rs17350579 (chr1:172.07 Mb). Both the minimum critical region identified by linkage analysis and the homozygous region were flanked by markers rs2758684 (chr1:157.54 Mb) and rs859652 (chr1:172.65 Mb) and include 15.12 Mb of sequence (Fig. S2b).

A DNA sample from the proband III-1 (Fig. 1a) underwent exome sequencing. Within the homozygous region on chromosome 1q23.1–q24.3, from the exome sequence data there are 14 homozygous variants with zero MAF in the ExAC database. Of the 14 rare variants, 13 variants were intergenic or intronic and occurred in dbSNP, and are unlikely to be disease-causal. On the other hand, a duplication variant c.355_356dupG (p.Glu119Glyfs*8) within *ATF6* (NM_007348.3) was absent in both dbSNP and ~120,000 ExAC alleles. Additionally, the variant was not identified in 470 ethnically matched control chromosomes or in exome sequence data from 130 unrelated Pakistani individuals with non-ophthalmologic traits. Sanger sequencing of DNA samples from the remaining members of family MA28 confirmed co-segregation of the c.355_356dupG variant with ACHM (Fig. 1a, c). Two-point linkage analysis using genotypes for the c.355_356dupG variant yielded a statistically significant LOD score of 3.6 (*θ* = 0).

The duplication of guanosine within exon 5 of *ATF6* is predicted to alter the reading frame (Fig. S2c), thus resulting in the substitution of glutamic acid at position 119 with glycine followed by a frameshift. The variant introduces a premature nonsense codon before the amino acids encoding the basic region leucine zipper (BRLZ) and transmembrane (TM) domains (Fig. S3). Multiple sequence alignment of human ATF6 and related sequences from 96 species indicated that several amino acid residues within the BRLZ domain are highly conserved or identical across species (Fig. S3a). These residues are also identical or highly conserved in various human transcription factors, supporting the important role of the BRLZ domain in target gene activation (Fig. S3b). Molecular modeling at ≥90 % confidence revealed that wild-type ATF6 is identical in sequence with other transcription factors by 45 % at maximum, but the sequence identity is limited to the BRLZ domain (Fig. S3c–d). Comparison of wild-type and mutant models for ATF6 predicts removal of ordered structure particularly of the BRLZ and TM domains (Fig. S3d).

There are three alternatively spliced isoforms of human *ATF6* (Fig. 1d, S2c). Isoform AK290498 has 16 exons and encodes a 670-amino acid protein. Exon 16 is alternatively spliced in isoform AF005887 at the 3′UTR but has the same coding region as in isoform A. Isoform AB208929 has 14 coding exons and encodes a 577-amino acid protein (Table S1). RT-PCR performed on commercially obtained RNA samples showed expression of all three *ATF6* isoforms in human eye, particularly in retinal pigment epithelium cells (Fig. 1d). All three *ATF6* isoforms include exons 1–5 in sequence, thus the duplication variant is predicted to affect all three isoforms in the same manner (Fig. S2c).

**Fig. 2:**
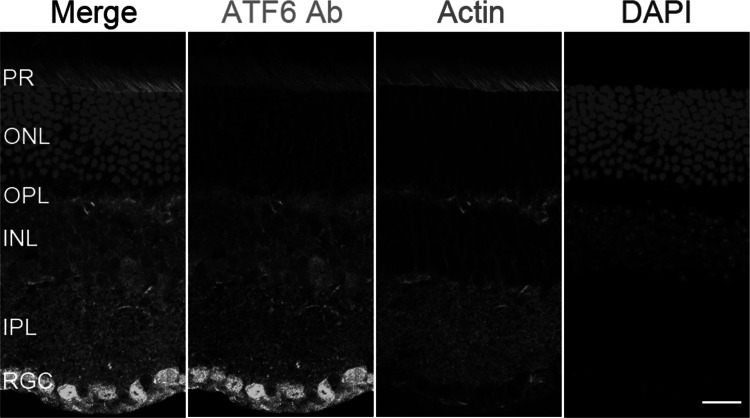
Localization of ATF6 in wild-type mouse retina. Immunostaining in CD1 mouse retina with antibodies for DAPI (*blue*), rhodamine phalloidin (*red*) for actin-based structures and ATF6 (*green*). ATF6 strongly localized to the retinal ganglion cells (RGC), and had moderate staining of the outer and inner segments of photoreceptor cells (PR) and the inner plexiform (IPL), inner nuclear (INL) and outer plexiform (OPL) layers. ATF6 is weakly localized to the outer nuclear layer (ONL) which contains photoreceptor cell bodies (color figure online)

The occurrence of multiple isoforms and ATF6 multimers in the eye is further confirmed by western blot analysis using anti-ATF6 antibodies on protein extract from adult C57BL/6J mouse retina (Fig. S3e). The anti-ATF6 antibodies were validated in heterologous cells. Immunofluorescent staining with anti-ATF6 antibodies overlapped with the signal produced by tagged ATF6 expressed in COS-7 cells (Fig. S4), which confirm the specificity of the antibody for ATF6. To explore the precise cellular localization in the eye, CD1 mouse retinae were examined for immunoreactivity with anti-ATF6 antibodies (Fig. 2, S5). ATF6 immunoreactivity is most prominent in the retinal ganglion cells. Staining of ATF6 was also observed in the retinal pigment epithelium, outer and inner segments of photoreceptor cells, inner and outer plexiform layers, as well as in the inner nuclear layer of neuronal retina (Fig. 2, S5).

**Fig. 3:**
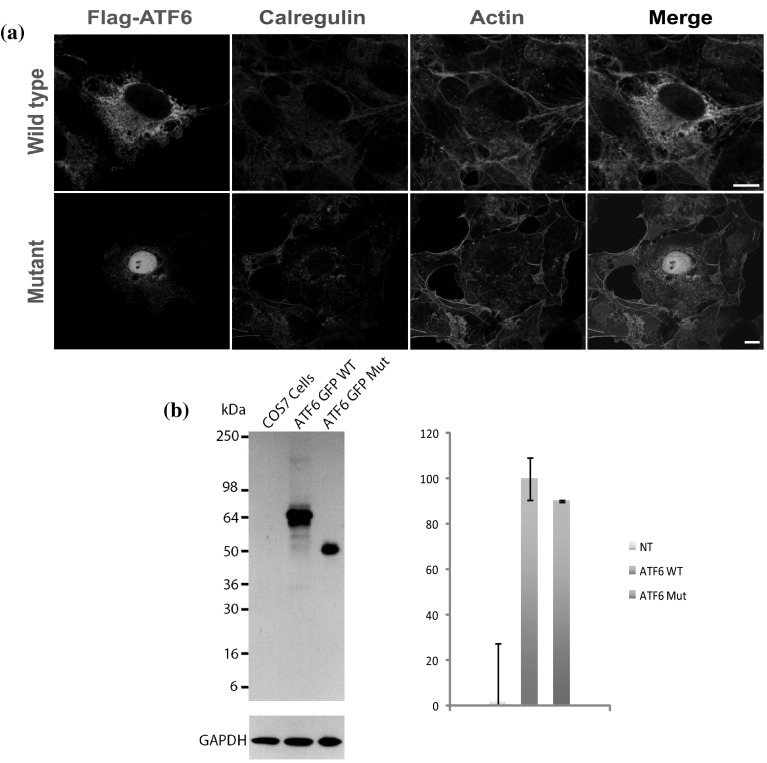
Expression of wild-type and mutant ATF6 in transfected COS-7 cells. **a** Flag-tagged wild-type ATF6 protein is expressed throughout the cytoplasm and localized in the endoplasmic reticulum. In contrast, the p.Glu119Glyfs*8 variant affected the stability, targeting and localization of ATF6, with expression confined to the nucleus and significantly reduced cytoplasmic localization. *Scale bars* 10 μm. **b** COS-7 cells were transiently transfected with the same quantity of wild-type or mutant *ATF6* constructs. Protein extracts from the cell lysates were analyzed by western blot using an anti-ATF6 antibody. Flag-tagged wild-type ATF6 protein is demonstrated at the expected size ~64 kDa and with dimer formation. In contrast, mutant ATF6 had reduced protein size. GAPDH antibody was used as loading control. Quantification of the steady-state expression did not reveal any significant difference between the wild-type and mutant ATF6

*ATF6* encodes a transcription factor that is initially synthesized as an ER-embedded transmembrane protein. To determine the effect of the ATF6 p.Glu119Glyfs*8 variant on steady state levels and localization, COS-7 cells were co-transfected with GFP-tagged human *ATF6* constructs. As expected, confocal imaging of the tagged wild-type ATF6 revealed expression throughout the cytoplasm and localization to ER (Fig. 3a). In contrast, mutant ATF6 with the p.Glu119Glyfs*8 variant had significantly reduced localization in the cytoplasm and was mainly confined to the nucleus (Fig. 3a). Western blot analysis and quantification, after normalization against the GAPDH expression level, revealed no significant difference in the steady state level of the p.Glu119Glyfs*8 mutant protein compared with wild-type ATF6 protein (Fig. 3b). Thus, in vitro, the p.Glu119Glyfs*8 variant affects the targeting and localization of ATF6.

## Discussion

In this study, we identified a rare variant in *ATF6* that segregates with the ACHM phenotype. *ATF6* lies within an autosomal recessive cone–rod dystrophy (CORD8) locus that was previously mapped in another Pakistani family to an 11.53-cM region on chromosome 1q12–q24 (Khaliq et al. 2000; Ismail et al. 2006). The affected individuals of the CORD8 family also presented with color vision loss, severe photophobia and progressive deterioration of vision since childhood. While the affected members of the CORD8 family had night blindness (Khaliq et al. 2000), the affected individuals from our ACHM family had normal night vision. Mutations in four previously identified ACHM genes namely *CNGA3*, *CNGB3*, *PDE6C* and *PDE6H* can cause both ACHM and CORD (Huang et al. 2013; Li et al. 2014b). However, negative results from Sanger sequencing of all exons of *ATF6* using DNA samples of two affected individuals from the CORD8 family ruled out the involvement of *ATF6* in the etiology of CORD. It is possible that another gene within chromosome 1q is responsible for CORD. On the other hand, genome-wide mapping and exome sequencing provided strong evidence that the *ATF6* c.355_356dupG (p.Glu119Glyfs*8) variant causes ACHM in family MA28.

Although ATF6 is ubiquitously expressed, *atf6*^−*/*−^ zebrafish and both *Atf6α*^−*/*−^ and *Atf6β*^−*/*−^ mice do not have obvious developmental abnormalities, while double knockout mice for *Atf6α*^−*/*−;^*Atf6β*^−*/*−^ were embryonic lethal (Wu et al. 2007; Yamamoto et al. 2007; Cinaroglu et al. 2011). There is also evidence that Atf6α-null mice subjected to high-fat diet develop glucose intolerance and after ischemic stroke have greater size of brain infarcts, both of which are explained by conditions of ER stress (Usui et al. 2012; Yoshikawa et al. 2015). In family MA28, there is no evidence of morphologic, cardiovascular, endocrine, or neurologic disease except for ACHM from early childhood. RT-PCR showed expression of all *ATF6* isoforms in retina (Fig. 1d), which are predicted to be equally affected by the *ATF6* c.355_356dupG variant. In humans, truncating *ATF6* variants that affect all isoforms may result in specific phenotypes brought about by ER stress, which in the case of the retina may be due to light exposure or inherited deficiencies (Thapa et al. 2012).

In general, cells respond to ER stress by activating the unfolded protein response (UPR) to facilitate ER protein folding and reduce misfolded protein levels (Chiang et al. 2012). In retinas exposed to ER stress due to excessive light injury, ATF6 mediates early UPR which serves to suppress ER stress and restore homeostasis (Nakanishi et al. 2013). ATF6 also activates IRE1 which mediates late UPR, during which ATF6 activation is decreased and severe or prolonged stress can result in apoptotic cell death (Lin et al. 2007; Nakanishi et al. 2013). ER stress-associated apoptotic cell death has been implicated as a common pathway for retinal degenerative disease, including ACHM due to gene defects in photoreceptor cyclic nucleotide-gated channels CNGA3 and CNGB3 (Thapa et al. 2012). Rod–cone photoreceptor dysfunction has been clearly demonstrated in the presence of mutation in other ACHM genes (Kohl et al. 2000; Thiadens et al. 2010; Aboshiha et al. 2014). Expression of previously identified ACHM genes has been equally distributed throughout the retina. Likewise, ATF6 has ubiquitous retinal expression with strongest localization in retinal ganglion cells and moderate staining at the retinal pigment epithelium, photoreceptor outer and inner segments, outer and inner plexiform and inner nuclear layers (Fig. 2, S5).

While all these cell layers that express ATF6 are widely known to be involved in color vision, absence of photopic a-waves in the ffERG of the ACHM proband from family MA28 supports functional loss of cone photoreceptors due to the *ATF6* duplication variant (Fig. 1b). The role of the ATF6 pathway in retinal ER stress response was previously described within photoreceptor cells (Yang et al. 2007, 2008; Saito 2014). In photoreceptors, ATF6 mRNA and protein expression is upregulated after light or drug exposure (Chiang et al. 2012; Li et al. 2014a). On the other hand, photopic b-wave diminution in the ffERG can be due to the absence of cone photoreceptor response and/or Müller and on-bipolar cell dysfunction at the inner retinal layers. Several studies have demonstrated the ER stress response through ATF6 within the inner retina. In particular, ER stress within normal and disease contexts (e.g., drug exposure, genetic loss of ER transmembrane protein, diabetic retinopathy) resulted in increased ATF6 expression in Müller glial and retinal ganglion cells (Yoshikawa et al. 2011; Wu et al. 2012; Miyagi et al. 2013; Ha et al. 2014; Zode et al. 2014). Although ffERG does not capture ganglion cell function, it is interesting to note that retinal ganglion cells primarily synapse with bipolar cells within the cone system. Retinal ganglion cells have been identified as the retinal cells most affected by ER stress due to diabetes (Yang et al. 2013) and glaucoma (Zode et al. 2014), and color vision defects have been documented in both acquired disease states (Lin and Yang 2010; Shoji et al. 2011). The unique retinal staining pattern of ATF6 with strongest localization to ganglion cells might imply that the ATF6 pathway within the retinal ganglion cells plays a major role in color vision.

In response to ER stress, ATF6 undergoes proteolysis to release its BRLZ domain, which binds to the ER stress response element to activate expression of downstream target genes within the nucleus (Haze et al. 1999). The interplay between transcription factors and target genes involved in the ER stress pathway is thought to allow fine-tuning of the UPR response that is cell or tissue specific (Saito 2014). Although MutationTaster (Schwarz et al. 2014) predicts the *ATF6* frameshift variant to initiate nonsense-mediated decay, immunolocalization studies in COS-7 cells overexpressing the mutant *ATF6* cDNA construct revealed the production of a smaller protein that is predominantly mislocalized to the nucleus (Fig. 3). Reduced cytoplasmic expression of truncated protein is to be expected given the loss of the TM domain that anchors ATF6 to the ER (Haze et al. 1999). While ATF6 is required to translocate to the nucleus to activate ER stress genes, the lack of a functional BRLZ domain in the truncated ATF6 protein should preclude DNA binding and UPR activation. Our experiments therefore predict that in vivo ATF6 protein with the frameshift variant is not reduced in overall amount of expression but is truncated, mislocalized and non-functional.

In conclusion, we identified a variant in *ATF6* as the underlying cause of autosomal recessive achromatopsia. This finding lends further support to the importance of the ATF6 pathway in retinal function. ATF6 is localized throughout the retina, with strongest staining within retinal ganglion cells. Taken together, our findings imply that the ATF6 pathway is essential in color vision not just within photoreceptors but also within the inner retina, and is a potential target for treatment for either inherited or acquired retinal diseases.

### Electronic supplementary material

Supplementary material 1 (PDF 744 kb) **Fig. S1 Full field ERG data obtained for the ACHM proband who is homozygous for the** ***ATF6*** **variant.** Scotopic or rod responses were normal at <30 Hz and subnormal with flattening of a and b waves at 120 Hz. Oscillatory responses were also demonstrable during 30 Hz rod testing. Cone flicker response was absent with no distinct and regular sinusoidal waveforms

Supplementary material 2 (PDF 883 kb) **Fig. S2 Mapping of ACHM in family MA28 to 1q23.1-q24.3 which includes the** ***ATF6*** **gene. (a)** HomozygosityMapper result (default threshold 0.8) on the first panel showing a single homozygous region in chromosome 1 that is shared by affected but not the unaffected family members. The genome-wide multipoint LOD scores on the second panel also support the mapping of ACHM gene to chromosome 1. **(b)** The mapped 1q23.1-q24.3 region that is flanked by markers rs2758684 and rs859642 includes *ATF6*. **(c)** For all three isoforms of *ATF6*, the c.355_356dupG (p.Glu119Glyfs*8) variant is predicted to result in a frameshift at the beginning of exon 5 and premature protein truncation

Supplementary material 3 (PDF 3830 kb) **Fig. S3 The** ***ATF6*** **c.355_356dupG** **variant is predicted to remove the basic region leucine zipper (BRLZ) domain which is highly conserved across species and human transcription factors.** The BRLZ domain includes highly conserved residues based on multiple sequence alignment using **(a)** 96 non-human ATF6 and similar sequences and **(b)** 13 other human transcription factors. The *ATF6* c.355_356dupG (p.Glu119Glyfs*8) variant is predicted to result in **(c)** loss of the BRLZ and transmembrane (TM) domains and **(d)** a truncated protein with highly disordered structure. **(e)** Western blot showing expression of multiple ATF6 protein products in mouse eye

Supplementary material 4 (JPEG 1754 kb) **Fig. S4 Validation of anti-ATF6 antibody.** Immunofluorescence images of COS-7 cells transfected with an expression vector containing flag-tagged cDNA construct for full-length human ATF6 protein (*green*) and stained with anti-ATF6 antibody (*red*). Actin (*magenta*) and nuclei (*blue*) labeled with rhodamine phalloidin and DAPI, respectively. Anti-ATF6 antibody immunofluorescence overlapped with the signal produced by tagged full-length protein reflecting the specificity of anti-ATF6 antibody for ATF6. Scale bar: 10 μm, all panels

Supplementary material 5 (JPEG 6491 kb) **Fig. S5 Localization of ATF6 in wildtype mouse retina at lower antibody dilution.** Legend as in Fig. 2

Supplementary material 6 (DOCX 32 kb)

## Appendix

### University of Washington Center for Mendelian Genomics

Michael J. Bamshad^1,2^, Jay Shendure^1^, and Deborah A. Nickerson^1^, Gonçalo R. Abecasis^4^, Peter Anderson^1^, Marcus Annable^1^, Mallory Beightol^1^, Brian L. Browning^1^, Kati J. Buckingham^1^, Christina Chen^1^, Jennifer Chin^1^, Jessica X. Chong^1^, Gregory M. Cooper^5^, Colleen Davis^1^, Lindsay Felker^1^, Christopher Frazar^1^, David Hanna^1^, Zongxiao He^3^, Preti Jain^5^, Gail P. Jarvik^1^, Eric Johanson^1^, Goo Jun^4^, Martin Kircher^1^, Tom Kolar^1^, Suzanne M. Leal^3^, Daniel Luksic^1^, Margaret J. McMillin^1^, Sean McGee^1^, Brenton Munson^1^, Brian J. O’Roak^1^, Bryan Paeper^1^, Karynne Patterson^1^, Eric Phillips^1^, Jessica Pijoan^1^, Christa Poel^1^, Peggy D. Robertson^1^, Regie Santos-Cortez^3^, Tristan Shaffer^1^, Cindy Shephard^1^, Deborah L. Siegel^1^, Joshua D. Smith^1^, Jeffrey C. Staples^1^, Holly K. Tabor^1,2^, Monica Tackett^1^, Gao Wang^3^, and Qian Yi^1^

^1^University of Washington

^2^Seattle Children’s Hospital

^3^Baylor College of Medicine

^4^University of Michigan

^5^HudsonAlpha Institute of Technology
