# Supplementary figures and images for: Mutation of ATF6 causes autosomal recessive achromatopsia
Source: Hum Genet. 2015 Jun 11;134(9):941–50. doi: 10.1007/s00439-015-1571-4 (PMC4529463; doi:10.1007/s00439-015-1571-4)

# 0.09 Hz Rod ERG

Right

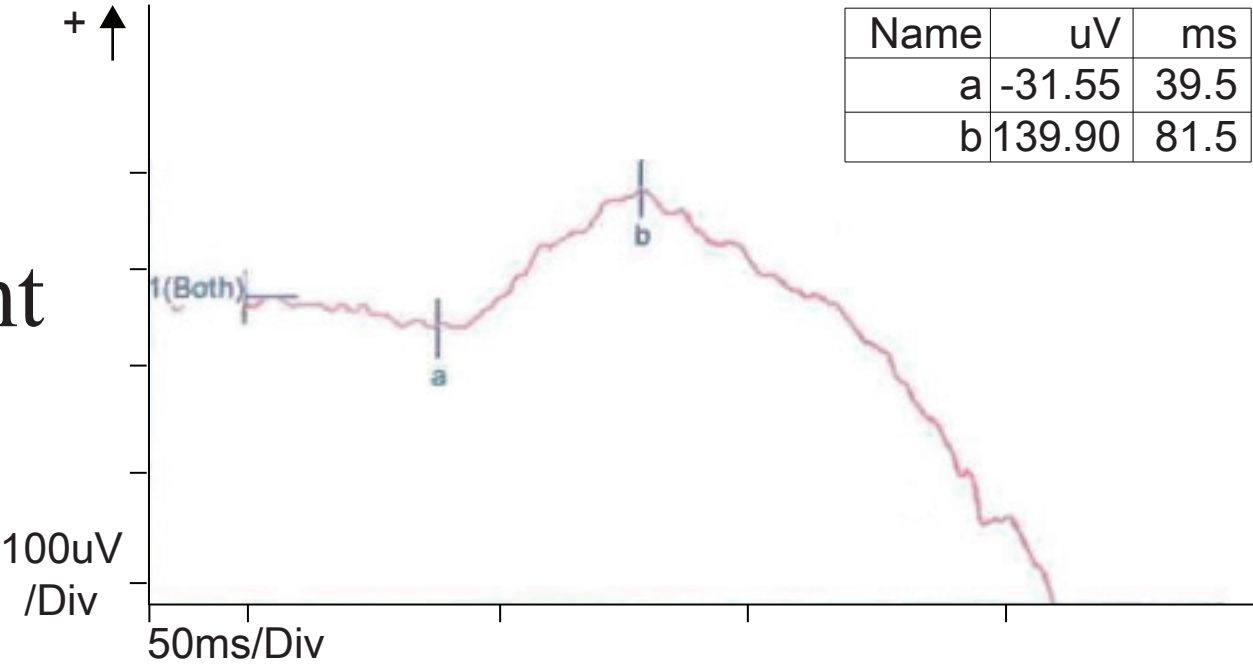

Left

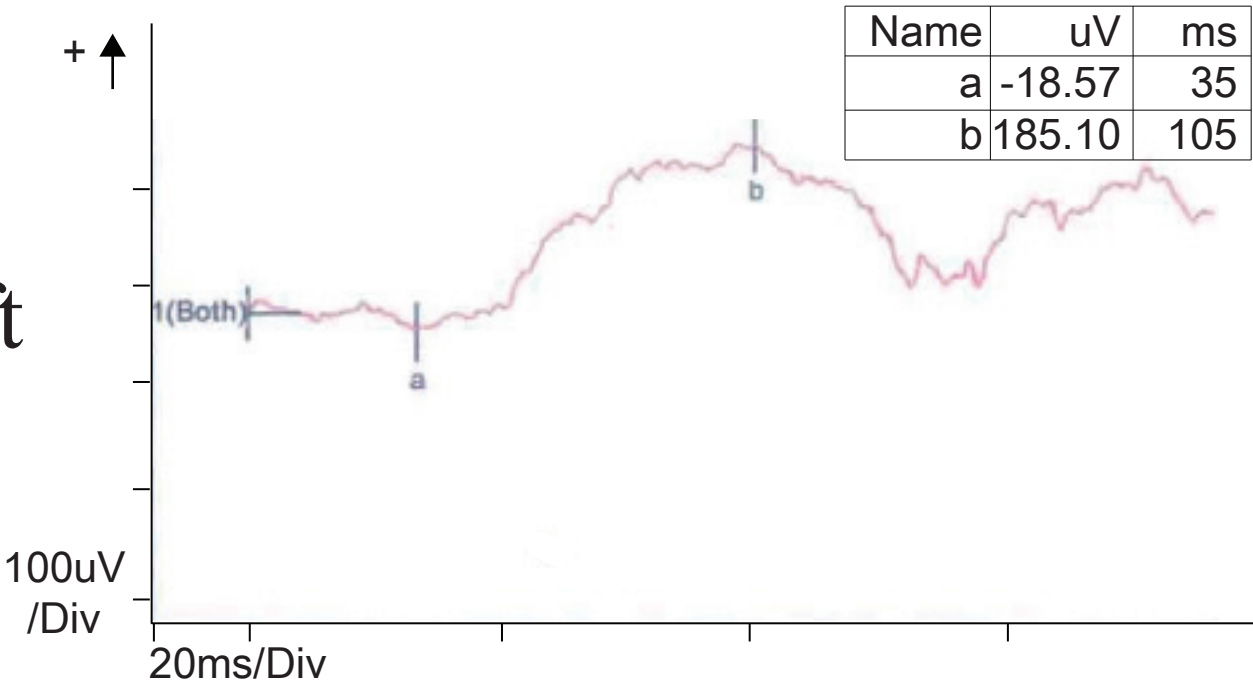

# 120 Hz Rod ERG

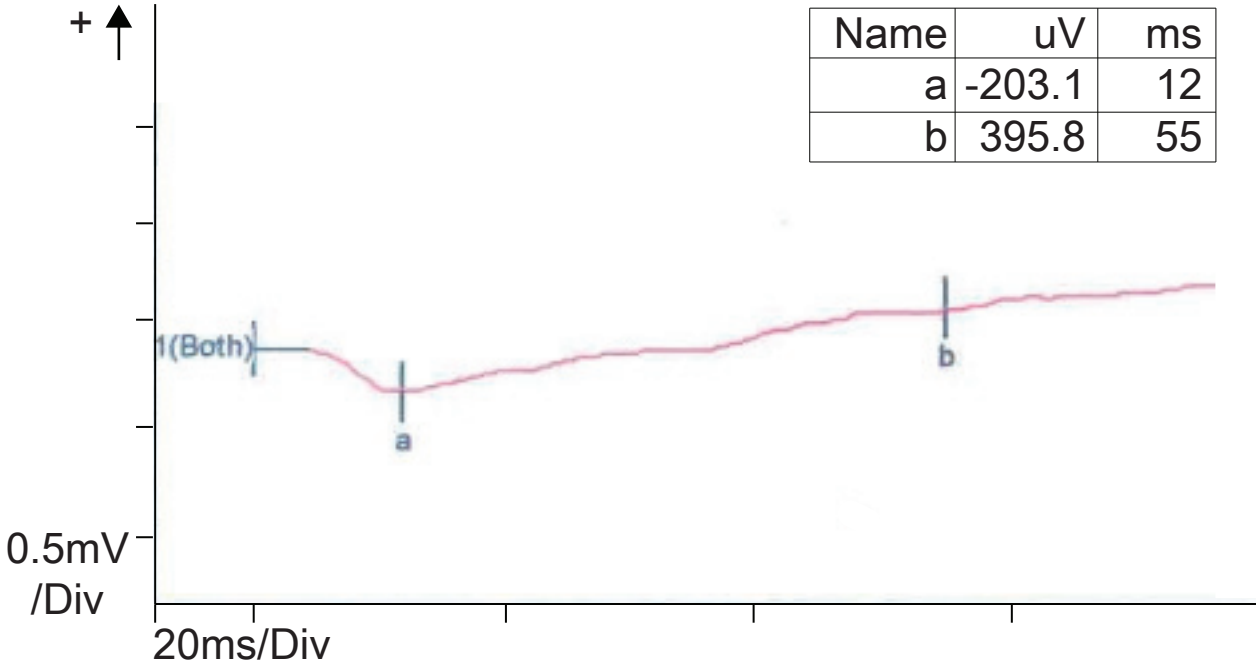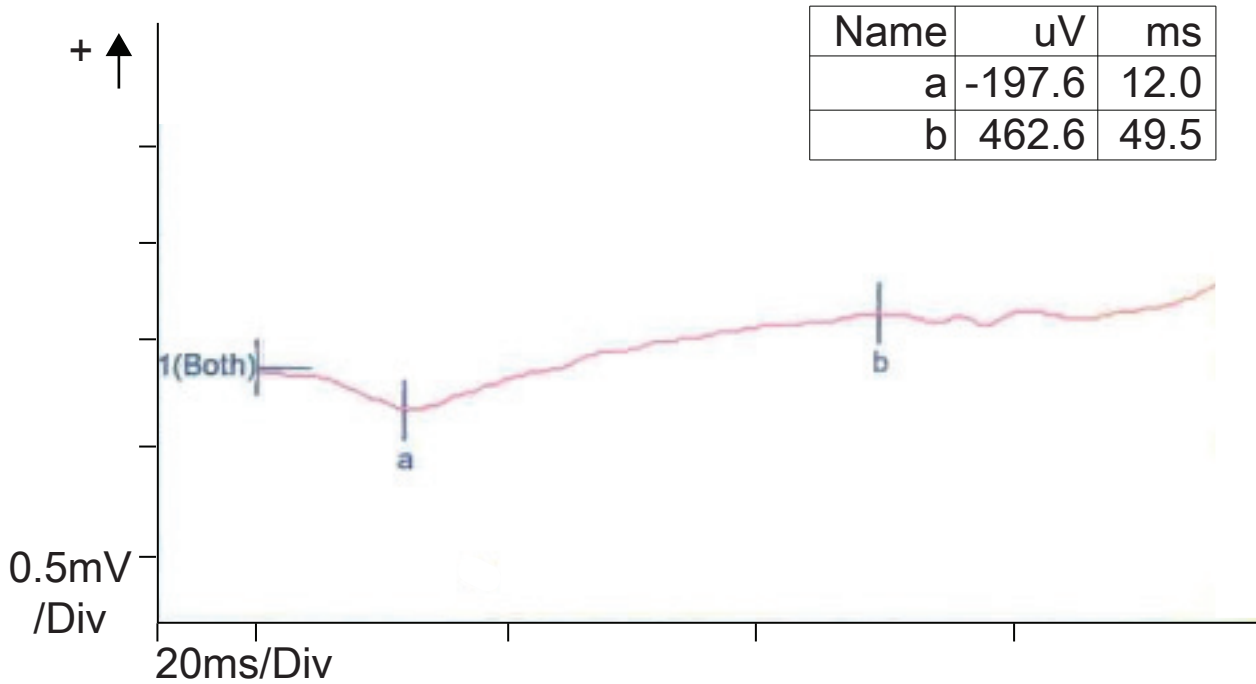

# Oscillatory Potential (Rod)

Right

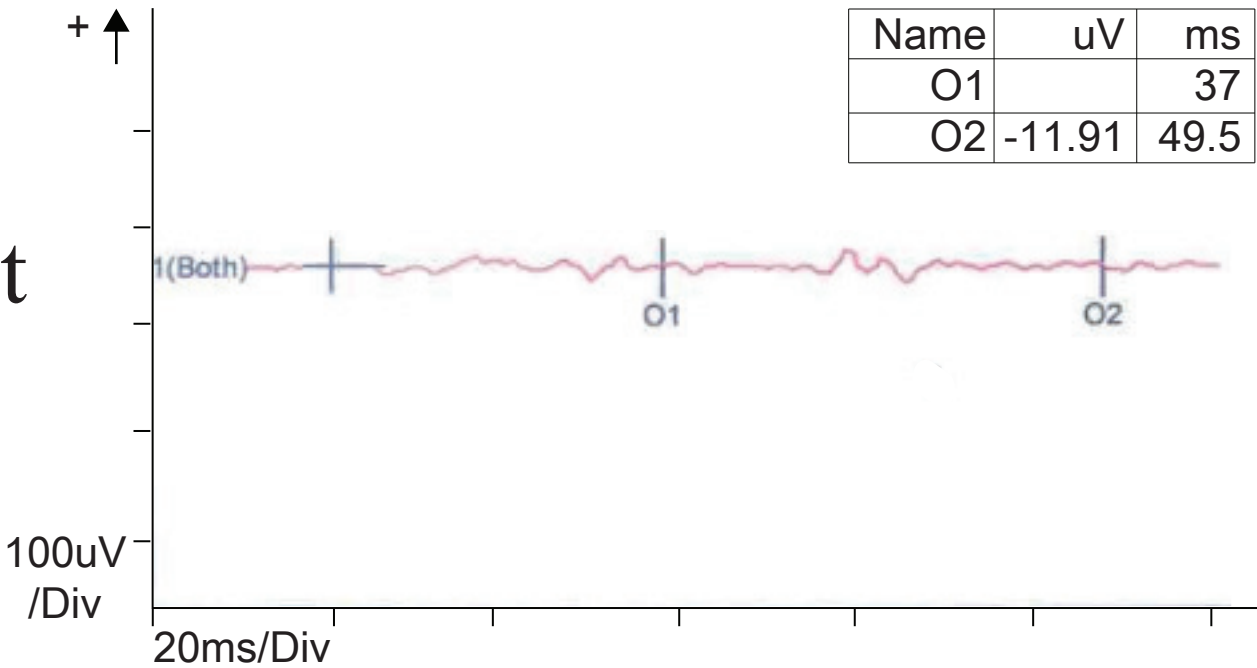

Left

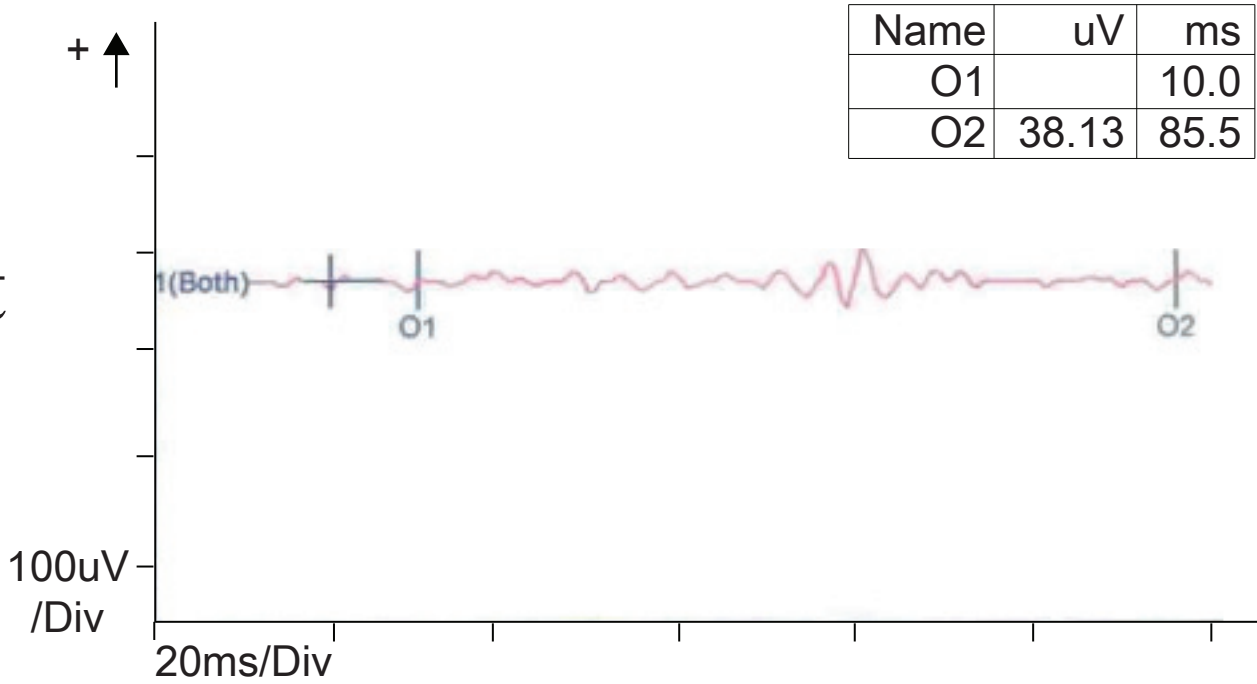

# Flicker Response

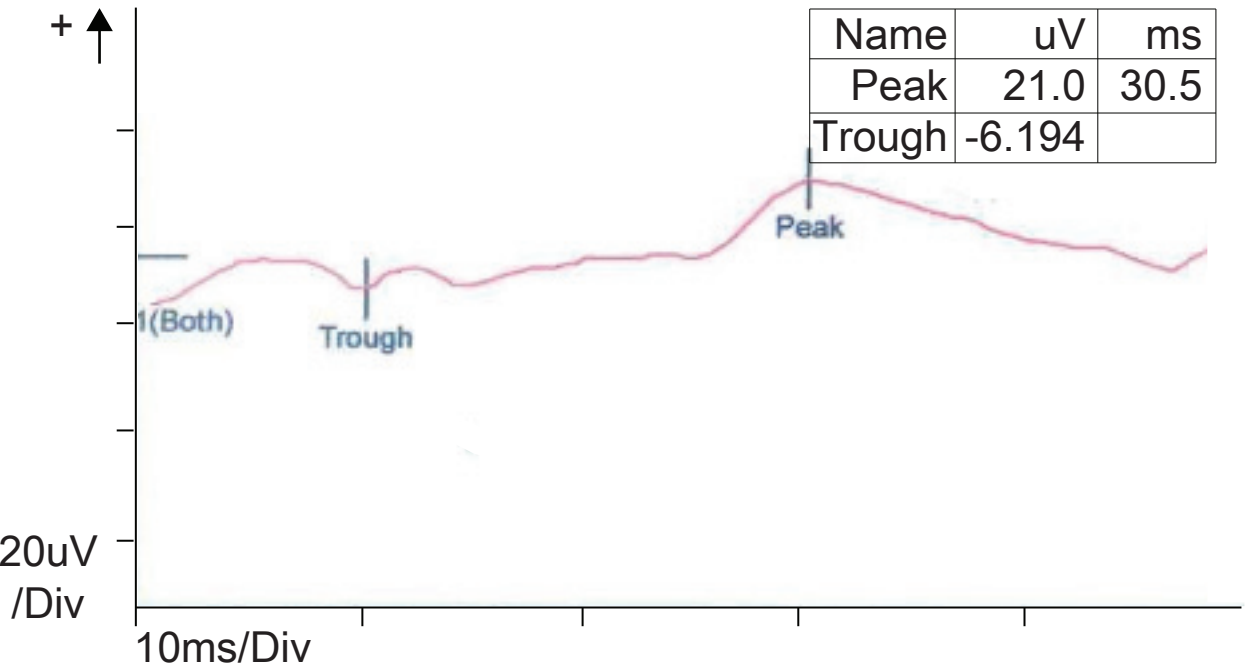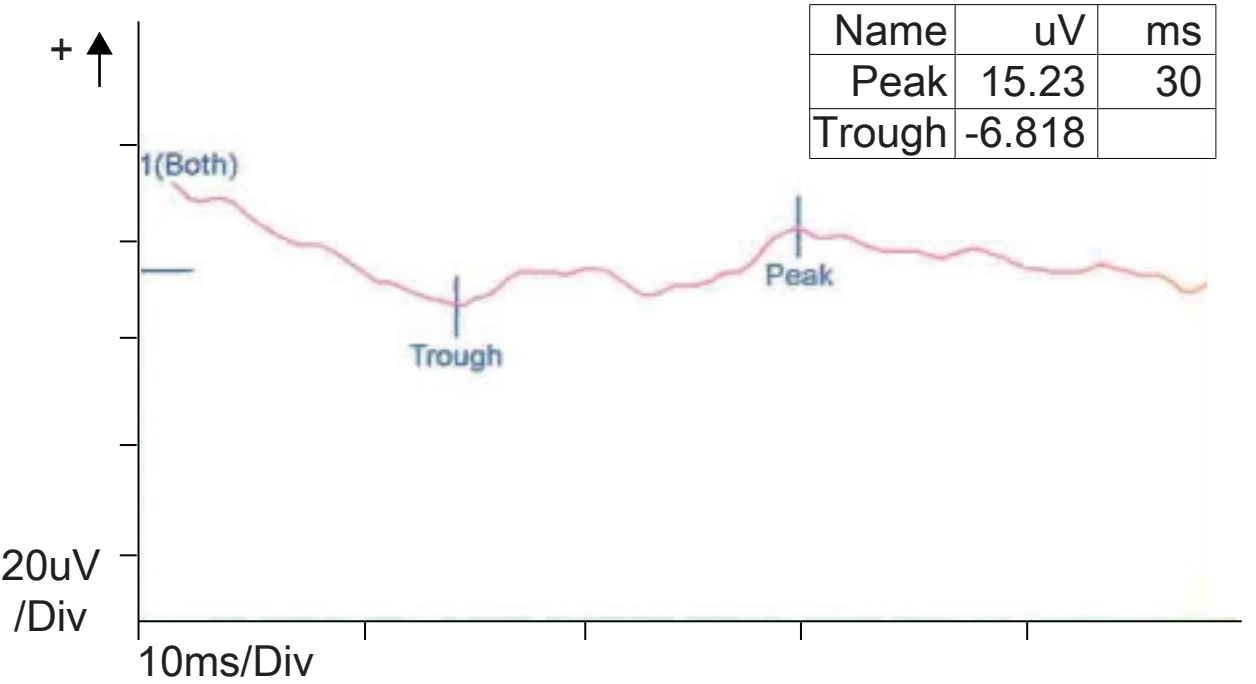

Supplement: Supplementary file 1 — Supplementary material 1 (PDF 744 kb) Fig. S1 Full field ERG data obtained for the ACHM proband who is homozygous for the ATF6 variant. Scotopic or rod responses were normal at <30 Hz and subnormal with flattening of a and b waves at 120 Hz. Oscillatory responses were also demonstrable during 30 Hz rod testing. Cone flicker response was absent with no distinct and regular sinusoidal waveforms [file 439_2015_1571_MOESM1_ESM.pdf]

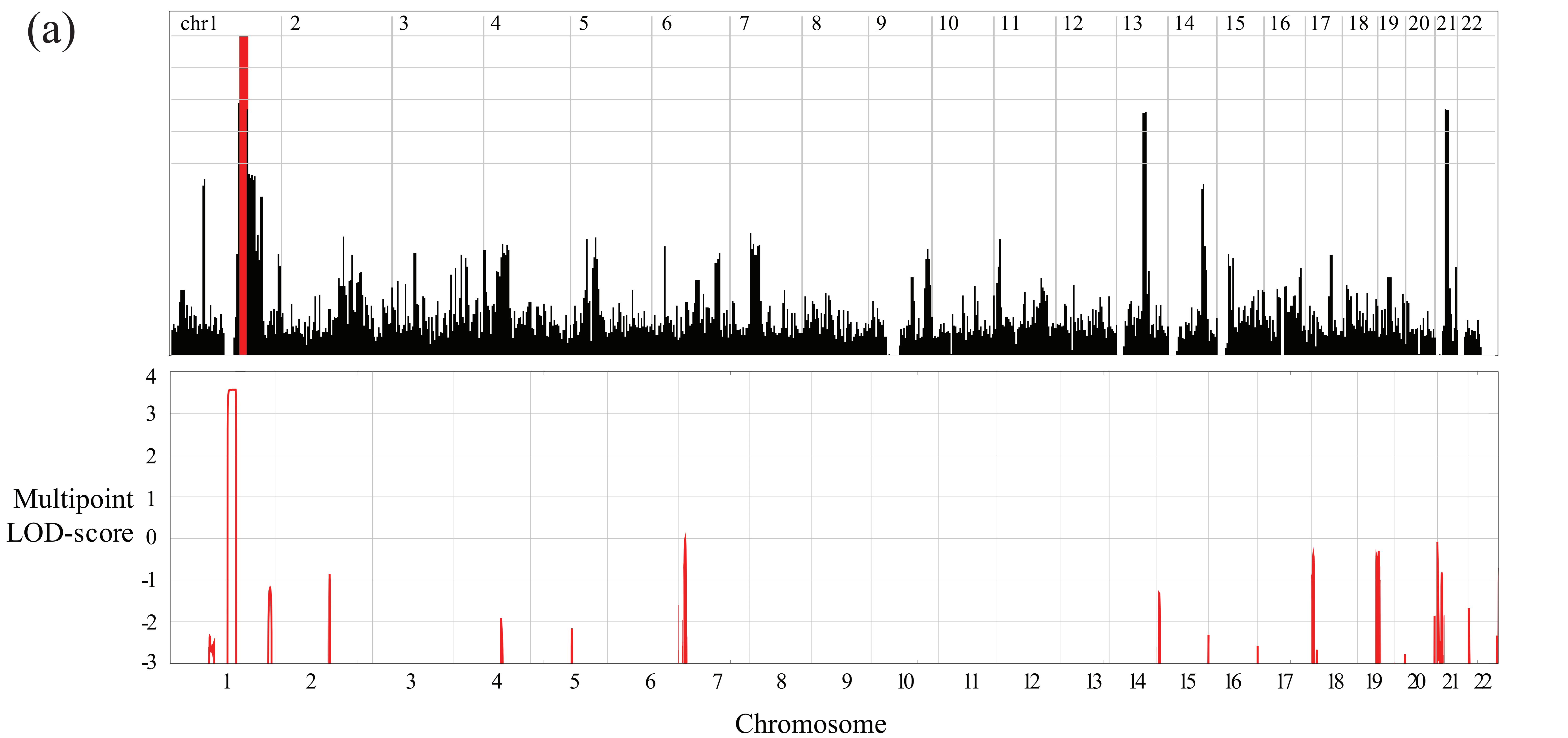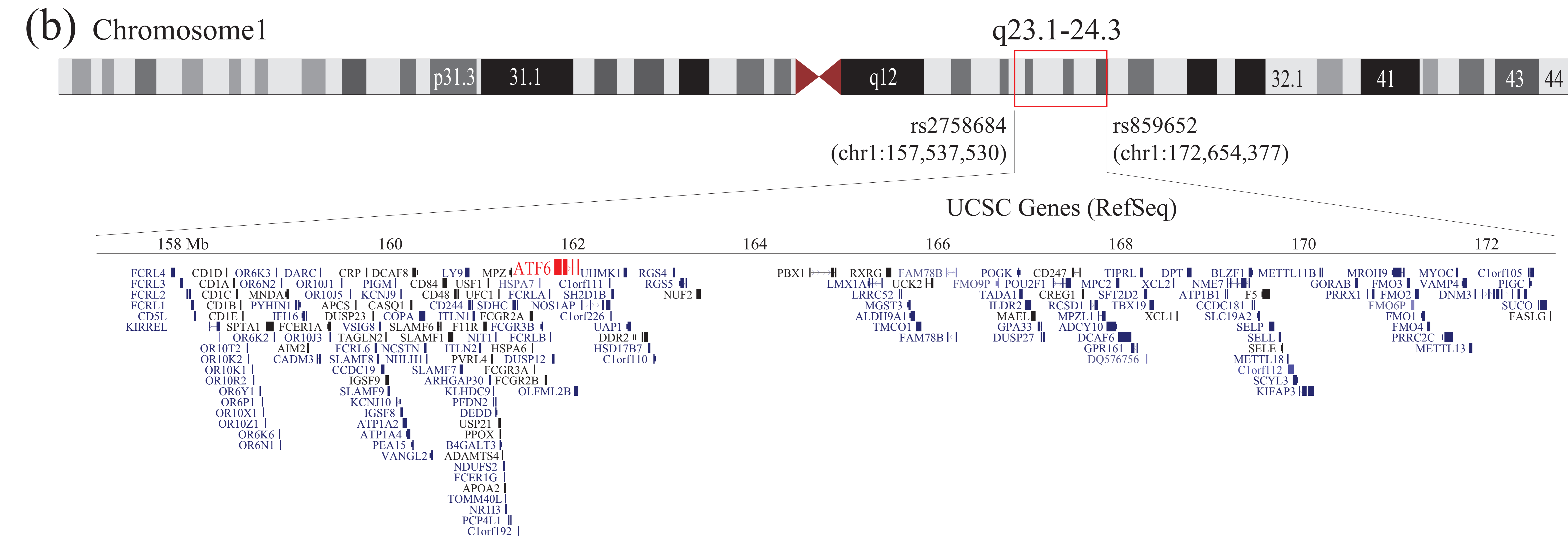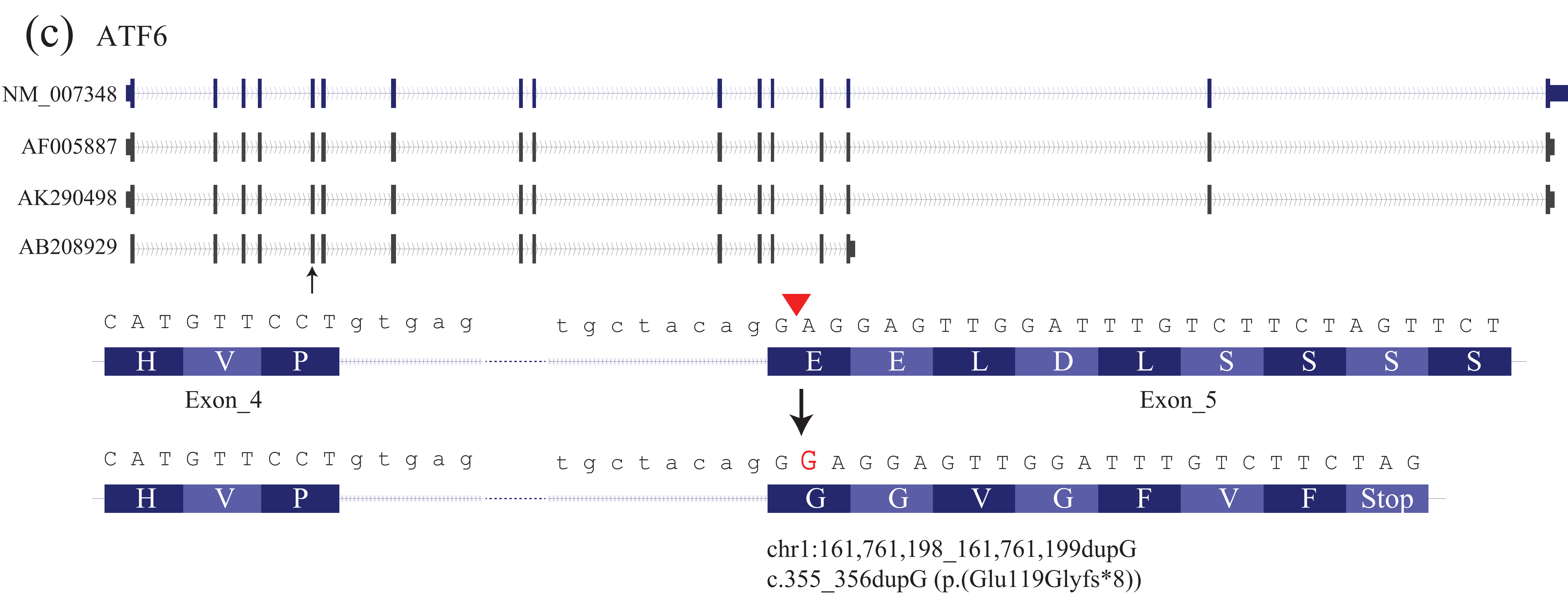

Supplement: Supplementary file 2 — Supplementary material 2 (PDF 883 kb) Fig. S2 Mapping of ACHM in family MA28 to 1q23.1-q24.3 which includes the ATF6 gene. (a) HomozygosityMapper result (default threshold 0.8) on the first panel showing a single homozygous region in chromosome 1 that is shared by affected but not the unaffected family members. The genome-wide multipoint LOD scores on the second panel also support the mapping of ACHM gene to chromosome 1. (b) The mapped 1q23.1-q24.3 region that is flanked by markers rs2758684 and rs859642 includes ATF6. (c) For all three isoforms of ATF6, the c.355_356dupG (p.Glu119Glyfs*8) variant is predicted to result in a frameshift at the beginning of exon 5 and premature protein truncation [file 439_2015_1571_MOESM2_ESM.pdf]

(a)

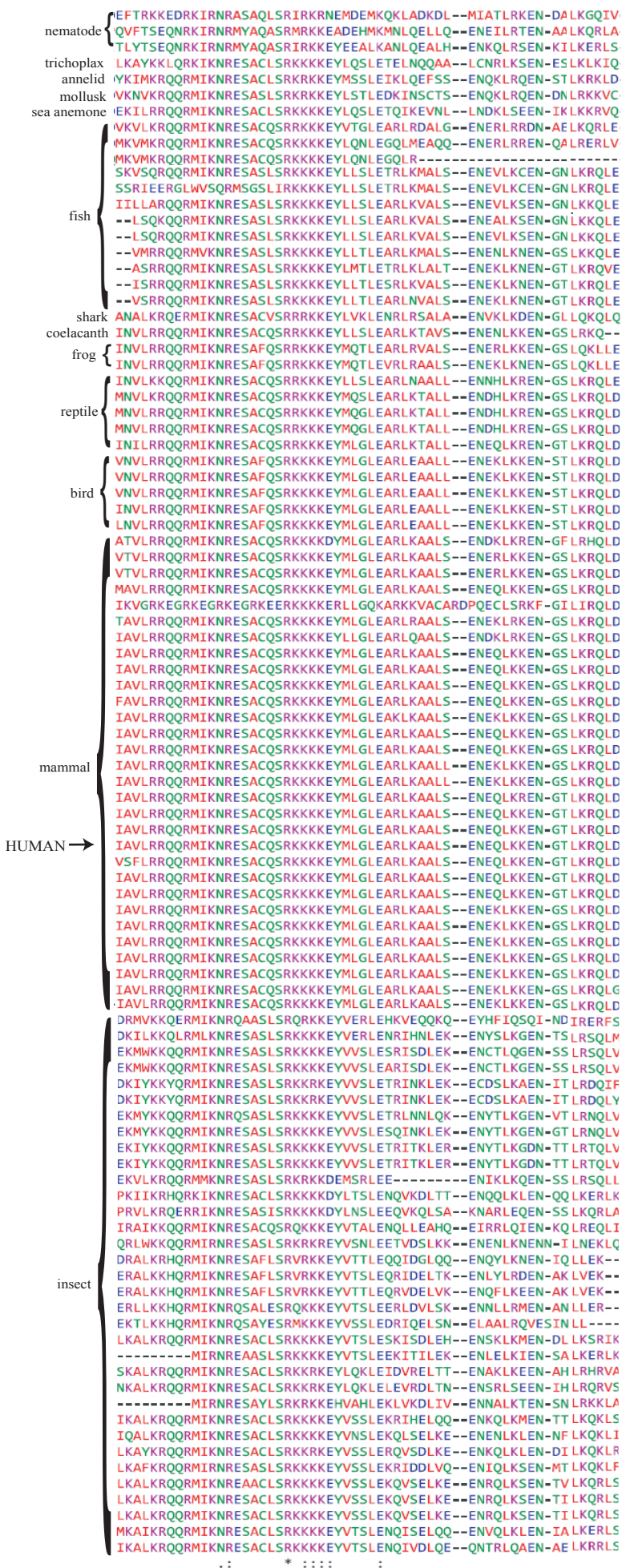

(b)

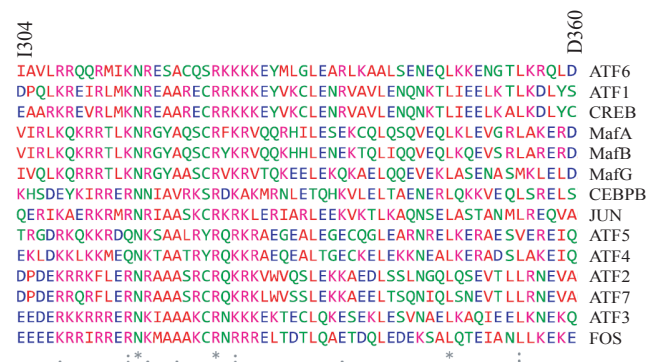

(c)

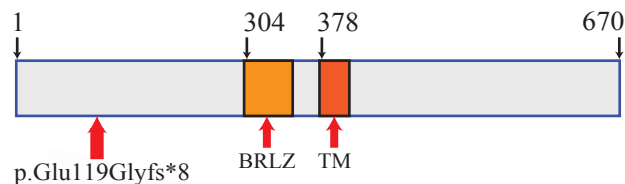

(d)

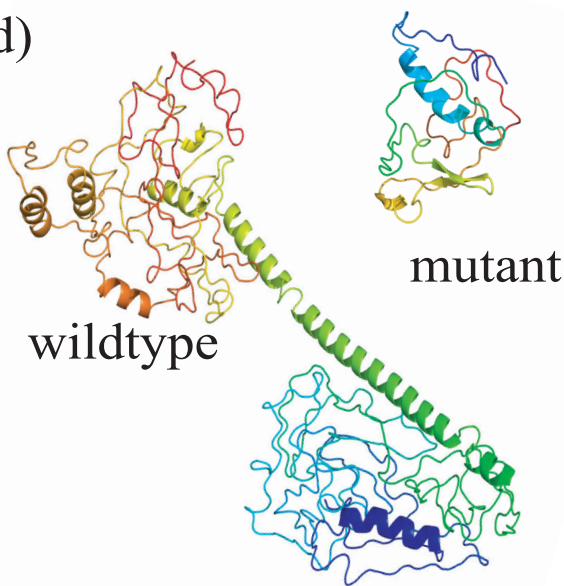

(e)

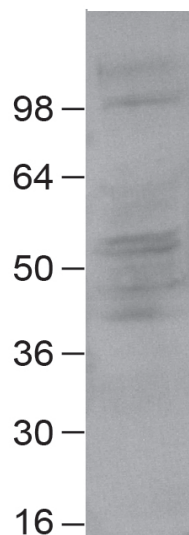

Supplement: Supplementary file 3 — Supplementary material 3 (PDF 3830 kb) Fig. S3 The ATF6 c.355_356dupG variant is predicted to remove the basic region leucine zipper (BRLZ) domain which is highly conserved across species and human transcription factors. The BRLZ domain includes highly conserved residues based on multiple sequence alignment using (a) 96 non-human ATF6 and similar sequences and (b) 13 other human transcription factors. The ATF6 c.355_356dupG (p.Glu119Glyfs*8) variant is predicted to result in (c) loss of the BRLZ and transmembrane (TM) domains and (d) a truncated protein with highly disordered structure. (e) Western blot showing expression of multiple ATF6 protein products in mouse eye [file 439_2015_1571_MOESM3_ESM.pdf]

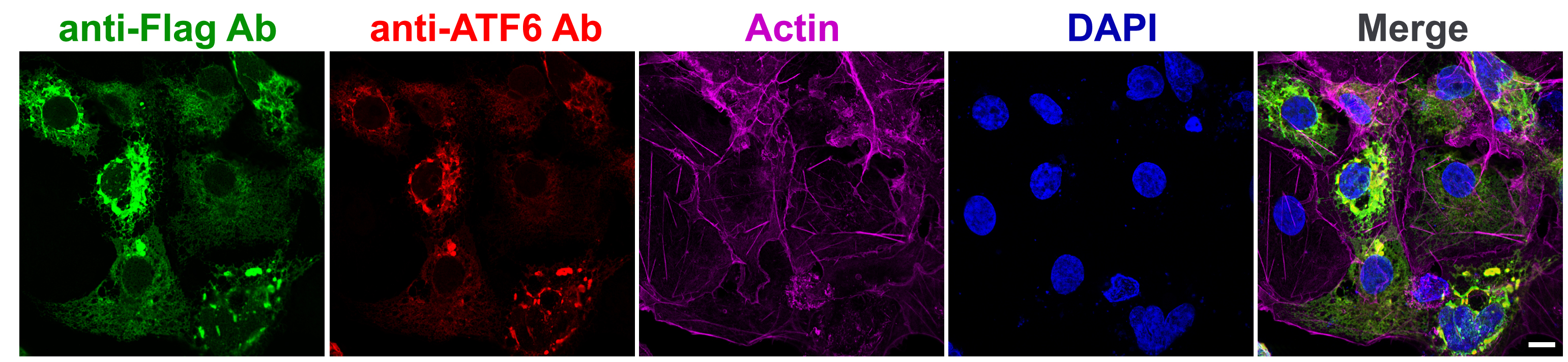

Supplement: Supplementary file 4 — Supplementary material 4 (JPEG 1754 kb) Fig. S4 Validation of anti-ATF6 antibody. Immunofluorescence images of COS-7 cells transfected with an expression vector containing flag-tagged cDNA construct for full-length human ATF6 protein (green) and stained with anti-ATF6 antibody (red). Actin (magenta) and nuclei (blue) labeled with rhodamine phalloidin and DAPI, respectively. Anti-ATF6 antibody immunofluorescence overlapped with the signal produced by tagged full-length protein reflecting the specificity of anti-ATF6 antibody for ATF6. Scale bar: 10 μm, all panels [file 439_2015_1571_MOESM4_ESM.jpg]

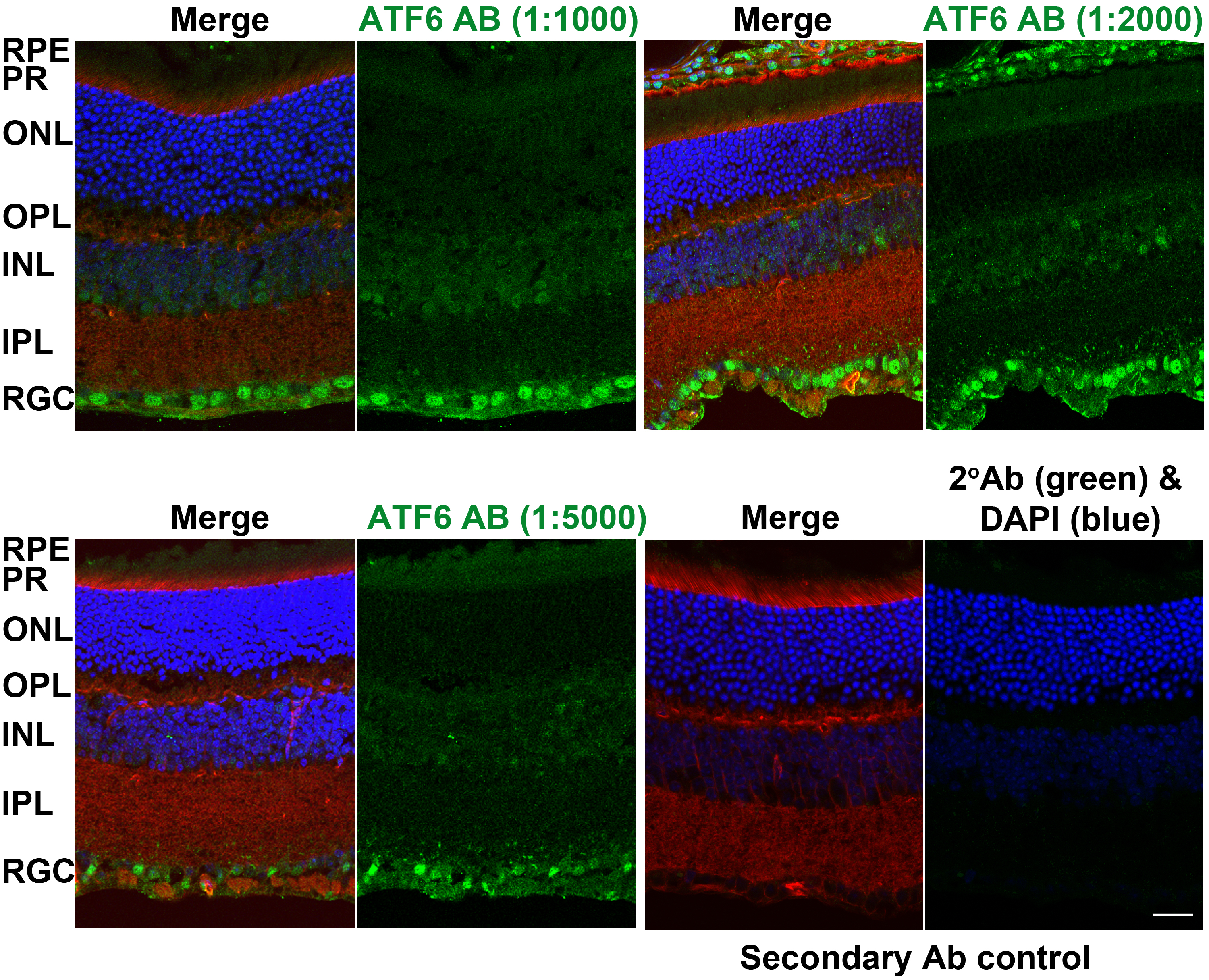

Supplement: Supplementary file 5 — Supplementary material 5 (JPEG 6491 kb) Fig. S5 Localization of ATF6 in wildtype mouse retina at lower antibody dilution. Legend as in Fig. 2 [file 439_2015_1571_MOESM5_ESM.jpg]
