# Supplementary material for: Mutation of ATF6 causes autosomal recessive achromatopsia
Source: Hum Genet. 2015 Jun 11;134(9):941–50. doi: 10.1007/s00439-015-1571-4 (PMC4529463; doi:10.1007/s00439-015-1571-4)
Supplement: Supplementary file 6 — Supplementary material 6 (DOCX 32 kb) [file 439_2015_1571_MOESM6_ESM.docx]

**Table S1 RT-PCR primers for *ATF6* isoforms and GAPDH**

| **Fig. 1 Label** | **Gene Isoform^a^** | **Target Exons** | **Forward Primer** | **Reverse Primer** | **Product Size (bp)** |
| --- | --- | --- | --- | --- | --- |
| I | NM_007348.3 | 1-16 | CCAGGGAGAAGGAACTTGTG | AATGACTCAGGGATGGTGCT | 2,028 |
| II | NM_007348.3 | 15-16 | CCACCCATAACAAGACCACA | AATGACTCAGGGATGGTGCT | 265 |
| III | AB208929 | 14 ^b^ | AAGGGACCTCACTGTGTTGC | GGCATCCTGCTAAGGTTGAG | 190 |
| GAPDH | GAPDH | 1-2 | ACATCGCTCAGACACCATG | TGTAGTTGAGGTCAATGAAGGG | 143 |

^a^ *ATF6* isoforms AK290498 and AF005887 have the same 16 coding exons as NM_007348.3. The two isoforms differ only by the 3’UTR. A third isoform AB208929 only has 14 exons.

^b^ The primers for AB208929 exon 14 were specifically designed to detect the longer coding region and UTR for this isoform. Exon 14 for NM_007348.3 only has 115 bp of coding region.
